# Multi-omics investigation of the resistance mechanisms of pomalidomide in multiple myeloma

**DOI:** 10.3389/fonc.2023.1264422

**Published:** 2023-09-19

**Authors:** Yan Zhuang, Chenyu Li, Hua Jiang, Lu Li, Yuanteng Zhang, Wei Yu, WeiJun Fu

**Affiliations:** ^1^State Key Laboratory of Genetic Engineering, School of Life Sciences, Fudan University, Shanghai, China; ^2^Department of Hematology, Shanghai Fourth People’s Hospital, School of Medicine, Tongji University, Shanghai, China; ^3^Institute of Drug Discovery and Design, College of Pharmaceutical Sciences, Zhejiang University, Hangzhou, Zhejiang, China

**Keywords:** multiple myeloma, pomalidomide, drug resistance, complement, glycine

## Abstract

**Background:**

Despite significant therapeutic advances over the last decade, multiple myeloma remains an incurable disease. Pomalidomide is the third Immunomodulatory drug that is commonly used to treat patients with relapsed/refractory multiple myeloma. However, approximately half of the patients exhibit resistance to pomalidomide treatment. While previous studies have identified Cereblon as a primary target of Immunomodulatory drugs’ anti-myeloma activity, it is crucial to explore additional mechanisms that are currently less understood.

**Methods:**

To comprehensively investigate the mechanisms of drug resistance, we conducted integrated proteomic and metabonomic analyses of 12 plasma samples from multiple myeloma patients who had varying responses to pomalidomide. Differentially expressed proteins and metabolites were screened, and were further analyzed using pathway analysis and functional correlation analysis. Also, we estimated the cellular proportions based on ssGSEA algorithm. To investigate the potential role of glycine in modulating the response of MM cells to pomalidomide, cell viability and apoptosis were analyzed.

**Results:**

Our findings revealed a consistent decrease in the levels of complement components in the pomalidomide-resistant group. Additionally, there were significant differences in the proportion of T follicular helper cell and B cells in the resistant group. Furthermore, glycine levels were significantly decreased in pomalidomide-resistant patients, and exogenous glycine administration increased the sensitivity of MM cell lines to pomalidomide.

**Conclusion:**

These results demonstrate distinct molecular changes in the plasma of resistant patients that could be used as potential biomarkers for identifying resistance mechanisms for pomalidomide in multiple myeloma and developing immune-related therapeutic strategies.

## 1 Introduction

Multiple myeloma (MM) is a hematological malignancy characterized by the proliferation of terminally differentiated plasma cells in the bone marrow (BM) (1). With more than 160,000 new cases diagnosed annually worldwide, MM is the second most common hematologic malignancy (2). Despite significant therapeutic advances over the last decade, MM remains an incurable disease (3).

Pomalidomide is a second-generation immune-modulatory drug (IMiD) that is commonly used to treat patients with relapsed/refractory multiple myeloma (RRMM). Compared to thalidomide and lenalidomide, pomalidomide exhibits superior anti-myeloma activity and immunomodulatory effects, significantly improving patients’ response rate and survival (4). Recent studies have identified Cereblon (CRBN) as a primary target of IMiDs anti-myeloma activity. Down-regulation of CRBN leads to drug resistance in MM cell lines and primary MM cells, while CRBN overexpression enhances sensitivity to IMiDs in myeloma cell lines. However, despite only 1.2% of MM patients exhibiting CRBN monoallelic deletion, a significant proportion of IMiD-refractory patients do not display detectable abnormalities of CRBN or its substrates. The single-agent response rate to IMiDs is about 30%, suggesting the existence of a CRBN-independent mechanism of IMiD resistance (5, 6).

As a class of anti-tumor agents, the research on pomalidomide has focused on not only their direct anti-proliferative activities against tumors, but also their ability to modulate the immune system. MM pathogenesis is linked to immune dysfunction, characterized by impaired T, B, and NK cells (5, 7). IMiDs have been shown to enhance the cytotoxicity of T and NK cells against MM cells. IMiDs activate T cell to produce cytokines such as interferon-γ (IFN-γ) and interleukin-2 (IL-2), which drive T cell clonal expansion (8, 9), and NK cell activation (10). T cell profiling studies in IMiD-treated MM patients consistently demonstrate a shift towards activated, proliferative, and cytotoxic T cell profiles (11, 12). Furthermore, IMiDs have varying modulatory effects on several immune cells, including dendritic cells and macrophages (13, 14). However, the mechanisms by which IMiDs activate T and NK cells remain incompletely understood. Recent studies have suggested that CRBN-mediated IKZF1/3 degradation in immune cells causes the depression of IL-2 and IFN-γ, leading to T and NK cell activation (15–17). IKZF1 degradation has also been implicated in the polarization of macrophages toward a proportion of tumoricidal M1 phenotype by IMIDs (14). However, only a proportion of IMiD-resistant cases are related to CRBN, and therefore additional mechanisms that are currently less well described need to be sought.

This study aimed to determine if there were molecular differences between patients who were resistant to pomalidomide and those who were sensitive. We performed proteomic and targeted metabolomic analyses to investigate molecular portraits of peripheral blood, followed by ssGSEA to estimate the differences in immune cellular composition. Additionally, we used WGCNA to evaluate the association between clinical cellular proportion features and modules of co-expression proteins. Our results demonstrate the distinct pattern of molecules in the plasma of resistant patients, which could aid in identifying resistance mechanisms for IMiDs and developing immune-related therapeutic biomarkers in MM.

## 2 Materials and methods

### 2.1 Ethics and human subjects

The study was executed in compliance with the Declaration of Helsinki and was approved by the medical Ethics Committee of the Shanghai Fourth People’s Hospital (2022186–001). All blood samples were collected before pomalidomide treatment. Collect peripheral blood from patients with heparin sodium blood collection vessels, centrifuge at 3000 rpm at room temperature, take the supernatant and divide it into centrifuge tubes, and quickly transfer it to −80°C refrigerator for storage. All study participants provided written informed consent and received detailed information on the study and associated risk prior to the enrollment.

### 2.2 Preparation of protein and peptide samples

Plasma samples were removed from -80°C and centrifuged at 4°C for 10 min at 12,000 g. Cell debris was removed and the supernatant was transferred to a new tube and removed using the High Abundance Protein Depletion Spin Columns Kit (Pierce™ Top 14 Abundant Protein Depletion Spin Columns Kit) (Thermo Scientific). Protein concentration was determined using the BCA kit. Then, 100 μg of plasma proteins per sample were reduced by 5 mM of dithiothreitol for 30 min at 56°C and alkylated by 11 mM of iodoacetamide for 15 min at room temperature in darkness. The alkylated samples were transferred to ultrafiltration tubes, centrifuged at 12000 g for 20 min at room temperature, replaced with 8 M urea three times, then replaced with urea three times with replacement buffer, added trypsin at a ratio of 1:50 (protease: protein, m/m), and digested overnight. The peptides were recovered by centrifugation at 12000 g for 10 min at room temperature, and then recovered by ultrapure water once, and the two peptide solutions were combined.

### 2.3 LC-MS/MS-based proteomic analysis

The peptides were separated by an UHPLC system and injected into an NSI ion source for ionization and then into an Orbitrap Exploris™ 480 mass spectrometer(Thermo Fisher Scientific) for analysis. The ion source voltage was set to 2.3 kV and the FAIMS compensation voltage (CV) was set to -45 V, -70 V. The peptide parent ions and their secondary fragment ions were detected and analyzed using a high-resolution Orbitrap. The primary mass spectrometry scan range was set to 400 - 1200 m/z, and the scan resolution was set to 60000; the secondary mass spectrometry scan range was fixed at 100 m/z, the secondary scan resolution was set to 30000, and TurboTMT was set to None. 15 peptide parent ions were sequentially entered into the higher energy collisional dissociation collision (HCD) cell (35% of collision energy), and the same sequential secondary mass spectrometry was performed. To improve the effective utilization of the mass spectrum, the automatic gain control (AGC) was set to 300%, the signal threshold was set to 1E4 ions/s, the maximum injection time was set to 100 ms, and the dynamic exclusion time of the tandem mass spectrometry scan was set to 30 s to avoid repeated scanning of the parent ions.

The secondary mass spectrometry data were retrieved using Proteome Discoverer (v2.4.1.15) for this experiment. Search parameters: Homo_sapiens_9606_SP_20200509.fasta (20366 sequences), inverse library was added to calculate the false positive rate (FDR) due to random matching; digestion mode was set to Trypsin (Full); number of missed cut sites was set to 2; minimum peptide length was set to 6 amino acid residues; maximum peptide Carbamidomethyl (C) was set as fixed modification, and Oxidation (M), Acetyl (N-terminus), Met-loss (M), Met-loss+acetyl (M) were set as fixed modification. loss+acetyl (M) were set as variable modifications. The FDR for protein, peptide, and PSM identification were all set to 1%.

### 2.4 Protein database search

Raw data were analyzed with Proteome Discoverer (v2.4.1.15) using the Andromeda database search algorithm (Thermo Fisher). The reference database contained 20,380 Swiss-Prot/reviewed human protein sequences downloaded from the UniProt database, and reverse decoy sequences were generated. Then, spectra files were searched against the merged database using the following parameters: Type, TMT; Variable modifications, Oxidation (M), Acetyl (Protein N-term); Fixed modifications, Carbamidomethyl (C), TMTpro (peptide N-Terminus), TMTpro (K); Digestion, Trypsin (Full). The MS1 match tolerance was set as 10 parts per million (ppm); the MS2 tolerance was set as 0.02 Da. Search results were filtered with 1% false discovery rate (FDR) at both protein and peptide levels. Proteins denoted as decoy hits, or only identified by sites were removed, and the remaining proteins were used for further analysis.

### 2.5 Targeted amino acid profiling

Amino metabolites were detected by HPLC-QqQMS/MS52. One hundred μL of the serum sample was mixed with 300 μL of cold methanol for protein precipitation. After 10 min of centrifugation (11060g, 4°C), 10 μL of supernatant was mixed with NEM solution for trapping thiols through click reaction forming RSH-NEM adducts. Then we removed multiple NEM by adding tBBT, and reduced disulfide bonds by TCEP. Derivatization of amino metabolites by 5-AIQC, we could quantitative detect 5-AIQC-RSH-NEM adducts and 5-AIQC-RSH adducts in a single run. The mixed standards were derivatized by the same way. The UPLC-QqQ-MS/MS system was made up of an Agilent 1290 UPLC coupled to an Agilent 6460 triple quadrupole mass spectrometer equipped with an Agilent Jet Stream electrospray ionization (ESI) source (Agilent Technologies, Inc.Santa Clara, CA, USA). Chromatography was carried out with a C18 column (Agilent Zorbax Eclipse XDB-C18 Rapid Resolution HD, 2.1 × 100 mm, 1.8 μm). Solvent A is 100% ultrapure water/0.1% formic acid, and solvent B is methanol/0.1% formic acid. The solvent program is: 1% B (0–2 min), 1–3.8% B (2–4 min), 3.8–22% B (4–8 min), 22–25% B (8–12 min), 25–60% B (12–13 min), 60–80% B (13–13.51 min), and 80–95% B (13.51–16 min). The flow rate is 0.6 mL/min and column temperature is 50°C. The mass spectra of the 5-AIQC-tagged amino metabolites with MRM mode showed the derivatized group including ions at m/z 171 to identify. Data acquisition and analysis were performed with Mass Hunter software (B.08.00, Agilent Technologies, Inc. Santa Clara, CA). The concentrations of amino metabolites were calculated from the calibration curves were prepared by diluting mixed standards.

### 2.6 DEP identification

T-test was used to identify DEPs between resistant patients and sensitive patients. DEPs with *P*< 0.05 and were considered statistically significant. The volcano map of DEPs was drawn using the “ggplot2” package.

### 2.7 Pathway enrichment analysis

GO and KEGG enrichment analyses was used to explore the potential biological functions of DEPs in resistant and sensitive patients. GO terms include biological process (BP), cellular component (CC), and molecular function (MF). The significance threshold of GO analysis was set as an adjusted value < 0.05, and KEGG analysis was set as value < 0.05. The above analysis results are presented as a bar chart and bubble chart by the “ggplot2” package.

### 2.8 Estimation of cellular proportion of immune cell types

We estimated the cellular proportions based on ssGSEA algorithm. The reference cell gene expression profile was obtained from transcriptomes data of sorted immune cells (18, 19).

### 2.9 Construction of gene co-expression network

The dataset was normalized and used for gene co-expression network construction via the weighted correlation network analysis (WGCNA) package (20). Pearson’s correlation coefficients were calculated between each pair of the proteins to generate the adjacency matrix. Then, the function “tomlikeity” was used to transform the adjacency matrix into the topological overlap measure (TOM). The TOM reflected the correlative interconnectivity between two proteins according to their degree of shared adjacency for the whole network. The proteins with similar expression patterns were clustered into the same module utilizing the average linkage hierarchical clustering based on the TOM-based dissimilarity measure.

### 2.10 Cell lines and culture conditions

The human MM cell lines RPMI-8226, MM.1S and U266 were cultured in RPMI 1640 medium, supplemented with 10% FBS, 1% penicillin, and 1% streptomycin. Cells were kept at 37°C in an incubator with 5% CO2.

### 2.11 Cell proliferation analysis

To assay cell growth, MM cells were washed twice with PBS and plated onto 96-well plates at a density of 3,000 cells per well. Cell numbers were calculated by using a cell counting chamber every day for 5 days. To assay cell viability, MM cells were plated onto 96-well plates at a density of 3000 cells per well. Cell numbers were measured by using a Cell Counting Kit-8 (APExBIO) every day for 5 days. Each test was repeated 3 times.

### 2.12 Apoptosis assay

Cell apoptosis was analyzed by flow cytometry with a FITC-Annexin V Apoptosis Detection Kit (Yeasen Biotech). The FITC-Annexin V and propidium iodide were used for double staining in accordance with the manufacturer’s instructions, followed by Gallios flow cytometry (Beckman Coulter). Kaluza for Gallios acquisition software (Beckman Coulter) was used to acquire the data and at least 10,000 events were collected in each analysis. The results were analyzed using FlowJo v10.0.7 software. Each test was repeated three times.

### 2.13 Enzyme-linked immunosorbent assay

Human protein ELISA kits were used to detect and quantify plasma levels of complement proteins according to manufacturers’ instructions (mlbio ELISA Kit). 50 μL of plasma sample and standard dilutions were added to the precoated plates. 50 μL diluted Biotin-Conjugate was added, and the plates were then incubated at 37°C for 1h. After 5 times washing, 100 μL Streptavidin conjugated Horseradish Peroxidase (HRP) was added and incubated at 37 °C for 1 h. 100 μL of Substrate Solution was added and incubated at 37 °C for 10 min. Finally, we added 50 μL of Stop Solution and detected the OD values at 450 nm using microplate spectrophotometer (BIO-RAD, xMark). The determination of OD values from serial dilutions of the standard samples was used to generate a standard curve of each protein and the relative concentrations of samples were calculated.

## 3 Results

### 3.1 Study design and blood samples

**Figure 1:**
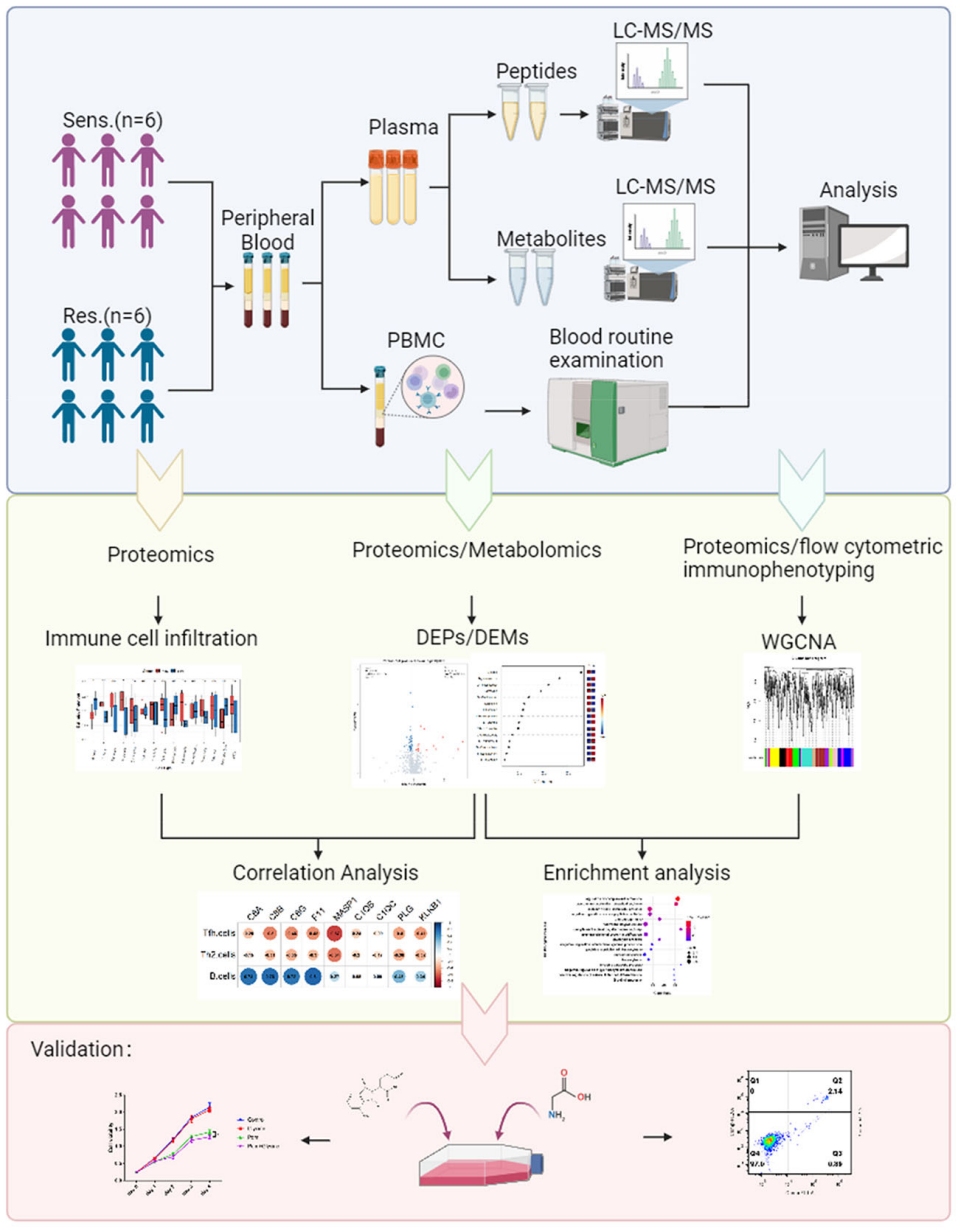
Study design and blood samples. Overview of plasma samples collected from pomalidomide resistant patients (n = 6) and pomalidomide sensitive patients (n = 6). The workflow for processing the proteomic and metabolomic data was shown, including the plasma separation, preparation of peptide samples, metabolite extraction, LC-MS/MS analysis, database search and further computational analyses.

From our cohort, we selected 12 relapsed/refractory multiple myeloma (RRMM) patients for whom peripheral blood samples were available prior to treatment. The clinical data of the 12 patients was shown (**Table 1**). These patients were segregated into two groups based on their response to pomalidomide and dexamethasone treatment: those who exhibited disease progression after 60 days of treatment were deemed resistant (n=6), while those who showed disease remission were deemed sensitive (n=6) (**Figure 1**). This rigorous and meticulous selection process ensured a high-quality dataset that enabled us to perform robust molecular and cellular analyses with the aim of uncovering the underlying mechanisms of pomalidomide resistance in MM.

**Table 1:** Patients' characteristics.

Clinical characteristics	Data	Sens.	Res.
Sex male/female n(%)			
male	9(75%)	3(25%)	6(100%)
female	3(25%)	3(50%)	0
Median age[years](range)	67.5 (54–76)	65.5 (60–75)	70.5(54-76)
MM types			
IgA	3(25%)	1(16.7%)	2(33.3%)
IgG	8(66.7%)	5(83.3%)	3(50%)
Light chain	1(8.3%)	0	1(16.7%)
Stage according to ISS n(%)			
I	1(8.3%)	1(50%)	0
II	6(50%)	3(50%)	3(50%)
III	5(41.7)	2(33.3%)	3(50%)
Cytogenetics n(%)			
high	6(50%)	3(50%)	3(50%)
non-high	6(50%)	3(50%)	3(50%)

### 3.2 Proteomic profiling reveals complement pathway and amino acid metabolism related to pomalidomide sensitivity

**Figure 2:**
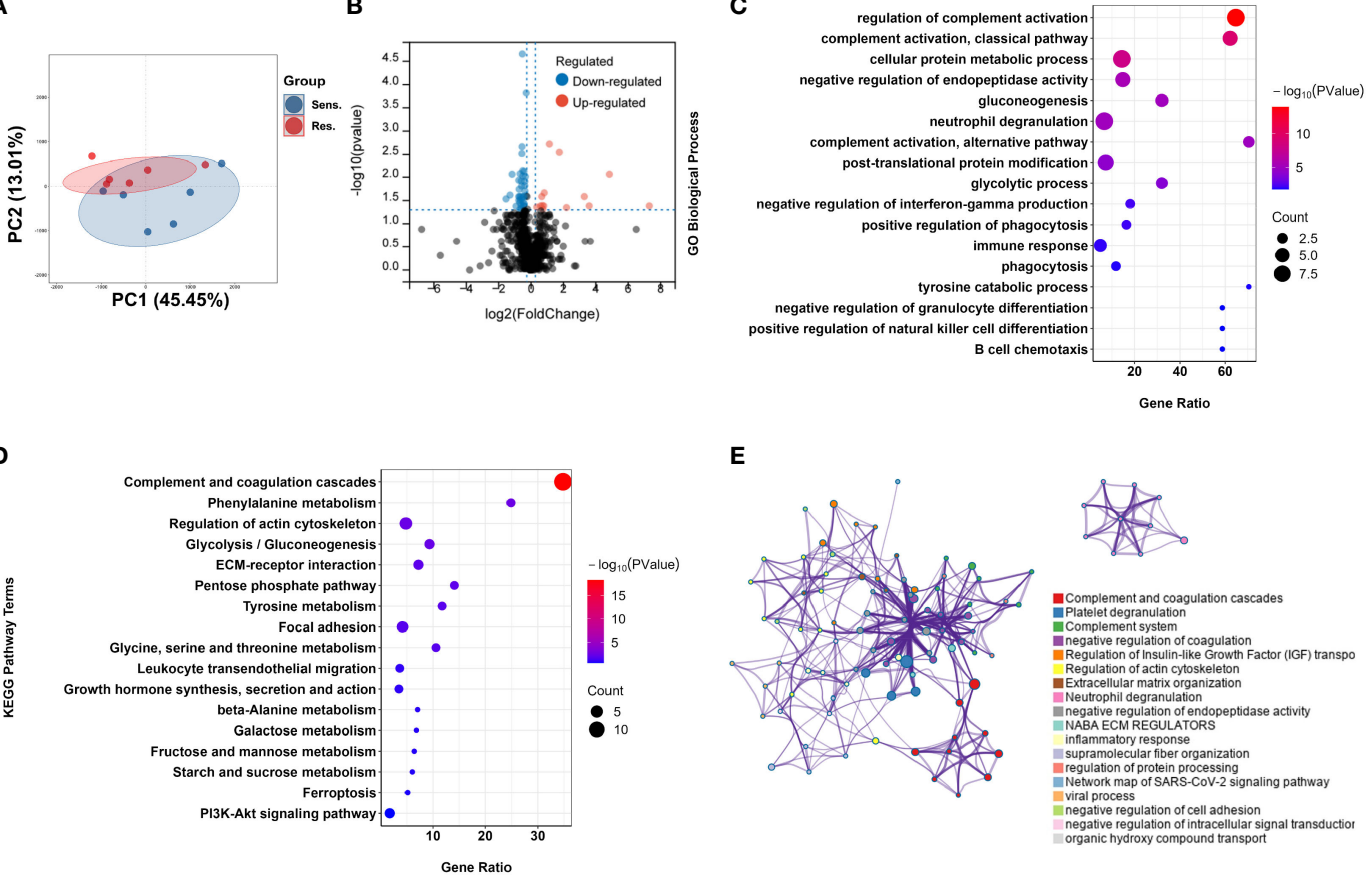
Proteomic analysis reveals a difference protein expression profiling between pomalidomide-sensitive and pomalidomide-resistant patients. **(A)** Principal component analysis (PCA) of protein expression in peripheral blood from MM patients. Each point represents one participant’s scores on the first 2 principal components (PC1 and PC2), where blue and red points correspond to sensitive and resistant group, respectively. Ellipses show the 80% confidence ellipse for each group. **(B)** Volcano plot for the comparison between the pomalidomide resistant and sensitive patients. The cutoff values of fold change ≥ 1.2 and *P* < 0.05 were utilized to identify differentially expressed proteins. Non-changed proteins are shown as gray dots, upregulated proteins as red dots, and downregulated proteins as blue dots. **(C)** GO-based enrichment analysis for DEPs of pomalidomide-resistant patients against pomalidomide-sensitive patients shown in the term of biological processes. **(D)** KEGG-based enrichment analysis for DEPs of pomalidomide-resistant patients against pomalidomide-sensitive patients. **(E)** Network plot of enriched ontology clusters colored by cluster ID, where terms with a similarity > 0.3 are connected by edges. A subset of enriched terms was selected based on the best p-values from each of the 20 clusters, and represented as a network plot, visualized by Cytoscape. Each node represented an enriched term and was colored by its cluster ID.

To investigate the underlying molecular mechanism of pomalidomide resistance in MM patients, we conducted a proteomic analysis of plasma samples from 12 MM patients, who were categorized into two groups based on their reactivity to pomalidomide treatment. A total of 1245 proteins were identified from all plasma samples. After filtering out the features with more than 80% NULL values, 849 protein groups remained for the following analysis. Using PCA analysis, we observed distinct distribution patterns between the pomalidomide-resistant group (n=6) and the pomalidomide-sensitive group (n=6) (**Figure 2A**). We identified 52 differentially expressed proteins (DEPs), including 15 up-regulated and 37 down-regulated proteins, with a P-value threshold of <0.05 and a fold change threshold of >1.2 (**Figure 2B**).

To further investigate the functions of these DEPs, we performed Gene Ontology (GO) and Kyoto Encyclopedia of Genes and Genomes (KEGG) pathway enrichment analyses. GO analysis revealed that the DEPs were significantly enriched in complement-related processes, including complement activation and regulation of complement activation, as well as immune response (GO:0006955), neutrophil degranulation (GO:0043312), B cell chemotaxis (GO:0035754), and tyrosine catabolic processes (GO:0006572) (**Figure 2C**). KEGG pathway analysis revealed that the DEPs were highly enriched in the Complement and coagulation cascades pathway (hsa04610), as well as in amino acid metabolism pathways such as Phenylalanine metabolism (hsa00360), Tyrosine metabolism (hsa00350), and Glycine, serine and threonine metabolism and amino acid metabolism (hsa00260) (**Figure 2D**).

**Figure 3:**
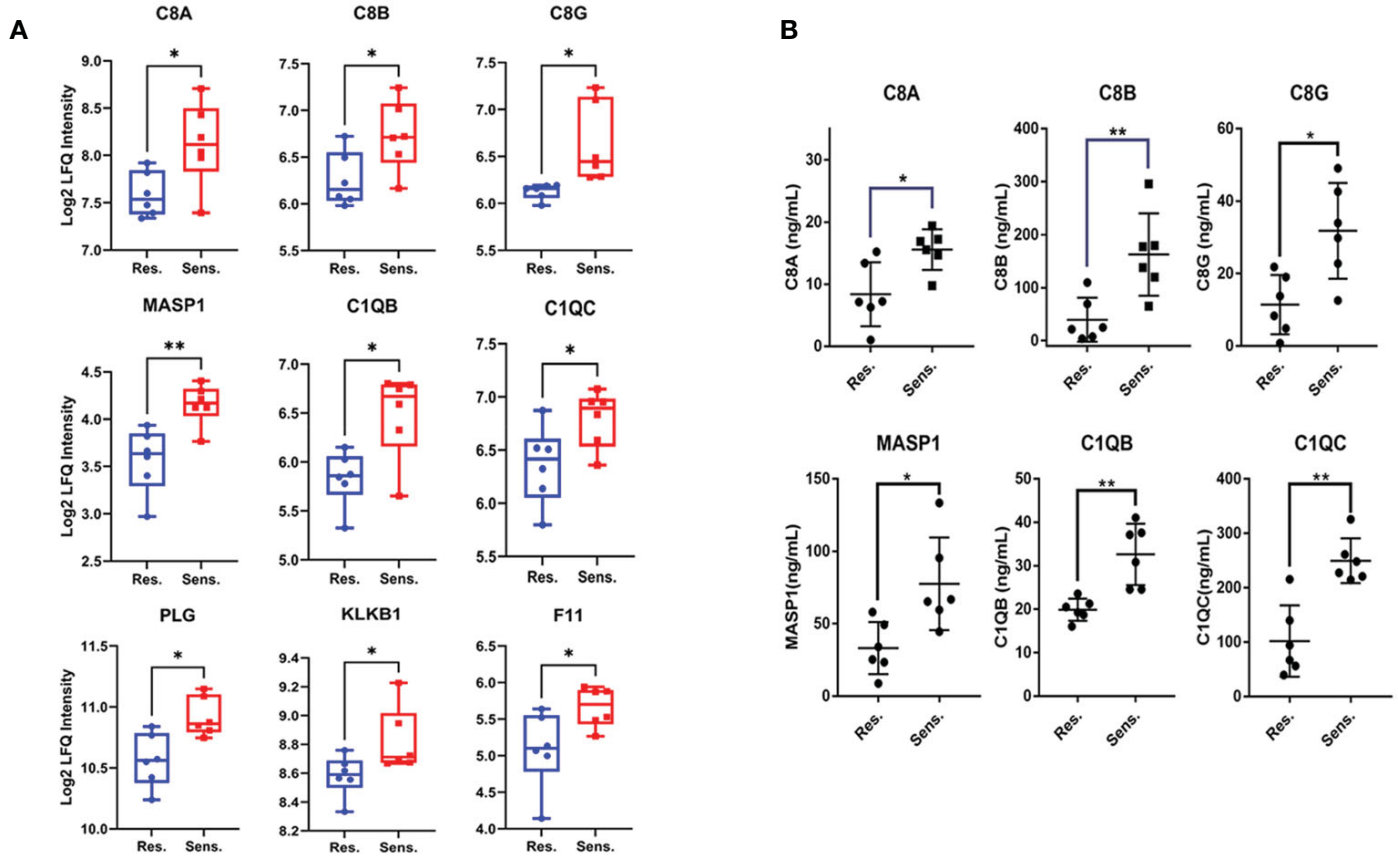
Validation of decreased protein expression of complement components in pomalidomide-resistant MM patients. **(A)** The expression levels of complement components in pomalidomide resistant and sensitive patients. **(B)** The expression levels of complements were validated via ELISA. The results were statistically analyzed by t-test. ns > 0.01, *P < 0.05, and **P < 0.01.

We further visualized the relationships between the enriched terms using Metascape analysis and found that the enriched terms were clustered into 18 distinct groups, with complement pathway and immune response being the most enriched clusters (**Figure 2E**). To validate the proteomic findings, we compared the levels of complement-related proteins, including MASP1, C1QB, C1QC, C8A, C8B, C8G, PLG, KLKB1, and F11, using ELISA. The results confirmed that pomalidomide-resistant patients had significantly lower levels of complement components than pomalidomide-sensitive patients (**Figures 3A, B**). The complement system is composed of three distinct pathways, including the classical (CP), lectin (LP), and alternative (AP) pathways, which can be activated depending on the context. The results of our study suggest that the complement pathway is significantly suppressed in pomalidomide-resistant MM patients. Specifically, C1Q and MASP are the recognition and activation molecules of the classic pathway and lectin pathway, while C8 participates in the formation of membrane attack complex (MAC) complexes during the terminal pathway of the complement system (21, 22). Additionally, PLG can activate the complement pathway independently of the classical and alternative pathways, while plasma kallikrein (KLKB1) is a serine protease that activates plasminogen (23). These results indicated that the complement pathway was significantly suppressed in the resistant group, providing insights for the development of novel therapeutic strategies.

### 3.3 Targeted metabolomic profiling reveals glycine related to pomalidomide sensitivity

**Figure 4:**
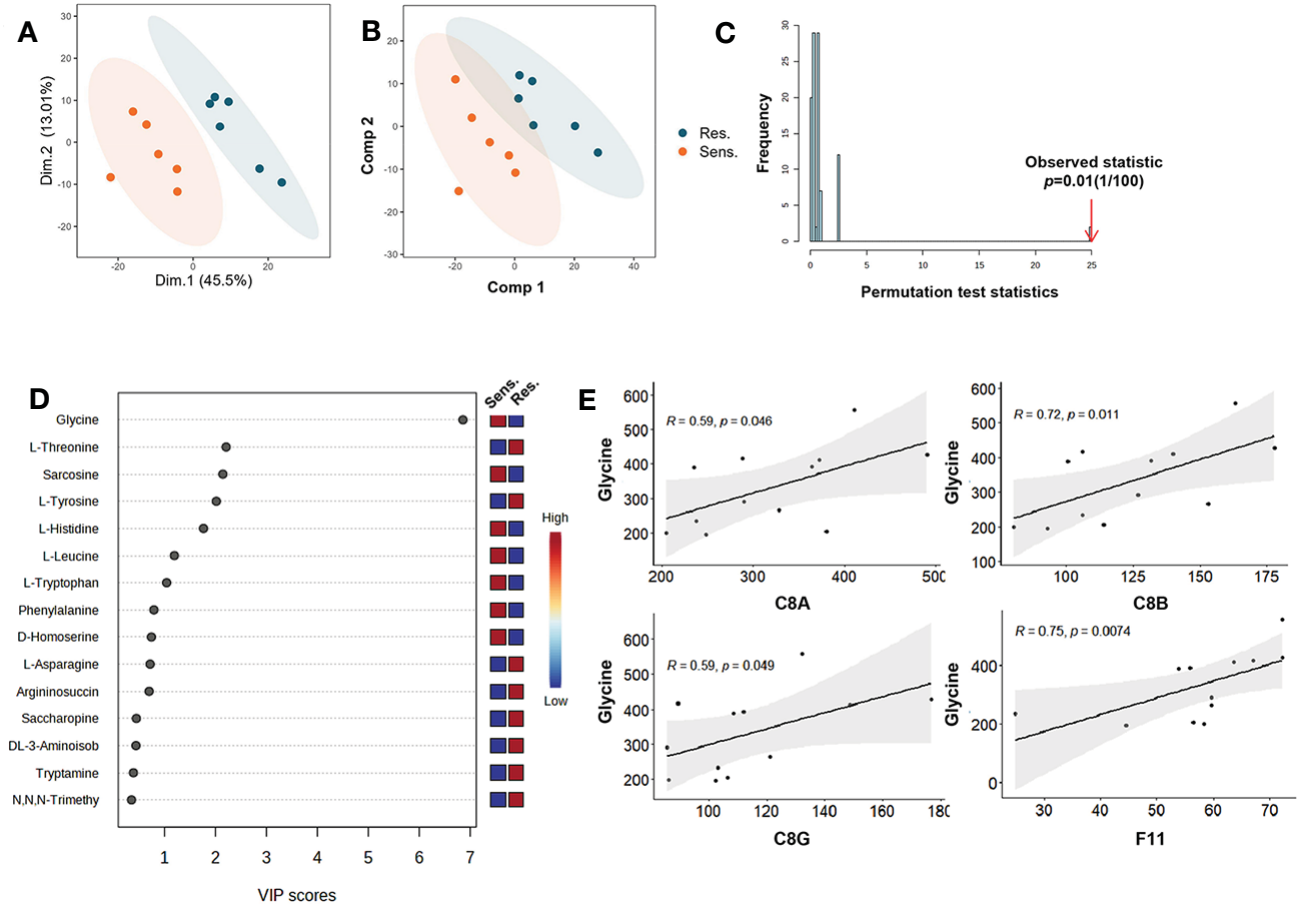
Targeted metabolomic profiling reveals glycine related to pomalidomide sensitivity. **(A)** PCA of metabolites in peripheral blood from MM patients. Each point represents one participant’s scores on the first 2 principal components (PC1 and PC2), where orange and purple points correspond to sensitive and resistant group, respectively. Ellipses show the 80% confidence ellipse for each group. **(B)** Partial least-square discriminant analysis (PLS-DA) of target Metabolomics by group. **(C)** Histogram of the permutation test. **(D)** VIP score plots of PLS-DA from sensitive and resistant group. **(E)** Correlation among the level of glycine and complement components. *P* < 0.05.

The fundamental role of amino acids in supporting the basic processes of life is widely recognized. In recent years, research has shown that amino acids also play a crucial role in supporting immune cell function, including ATP generation, nucleotide synthesis, and redox balance (24). Metabolomics is a powerful tool for investigating the metabolic changes and regulations of small molecules, has become increasingly important in understanding the role of amino acids in various physiological processes (25). In this study, we leveraged targeted metabolomics for amino acids to analyze plasma samples from patients with multiple myeloma to gain insights into the molecular mechanisms underlying resistance to pomalidomide therapy. Here, we used PCA and partial least square discriminant analysis (PLS-DA) to analyze the targeted metabolomics data and observed that good discrimination between the resistant and sensitive groups was attainable (**Figures 4A, B**, **S1**). The permutation test on the PLS-DA model further confirmed that the differences between the two clusters were significant, indicating that our model did not have overfitting (**Figure 4C**). Metabolites with a variable importance in the projection (VIP) value > 1 were considered to contribute to the classification of the two groups. Our results indicated that 7 amino acids were significantly associated with the classification (**Figure 4D**). The contribution of glycine was the most significant, and it showed a significant correlation with complement components (**Figure 4E**), suggesting a potential relationship between glycine and the complement pathway in the resistance response of multiple myeloma patients to pomalidomide.

### 3.4 Combination treatment of pomalidomide with glycine has additive apoptotic effects on MM cells

**Figure 5:**
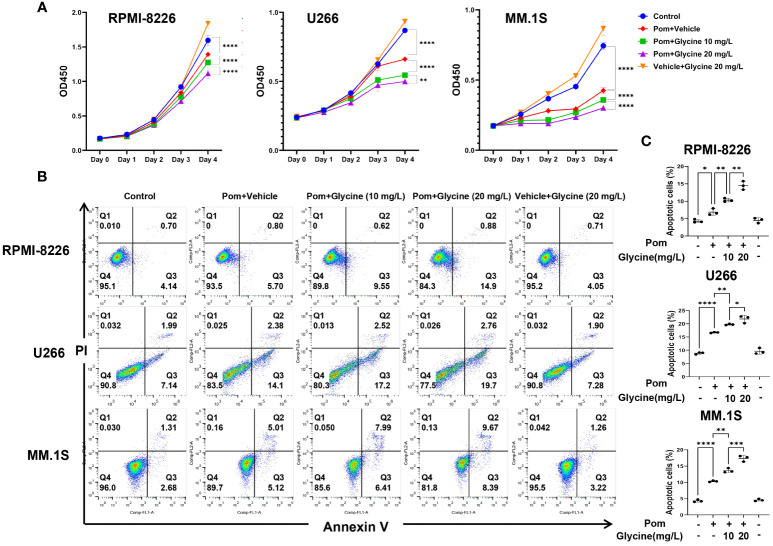
Combination treatment of pomalidomide with glycine has additive apoptotic effects on MM cells. **(A)** Cell viability of RPMI-8226, MM.1S and U266 cells upon 96 hours treatment with pomalidomide (1 μM) and different concentration of glycine (0, 10, 20 mg/L) or a combination thereof. (N = 3 biologically independent replicates) **(B)** Flow cytometric images to detect apoptosis and **(C)** percentage of apoptotic MM cells upon 96 hours treatment with pomalidomide (1 μM) and different concentration of glycine (0, 10, 20 mg/L) or a combination thereof. (N = 3 biologically independent replicates). *P < 0.05, **P < 0.01, ***P < 0.001, and ****P < 0.0001.

To investigate the potential role of glycine in modulating the response of multiple myeloma (MM) cells to pomalidomide, conducted *in vitro* experiments using MM cell lines. Specifically, we cultured MM cells in glycine-free RPMI 1640 media supplemented with different concentration of glycine (0, 10, 20 mg/L) and assessed their response to pomalidomide. Our results demonstrated that the addition of exogenous glycine increased the sensitivity of MM cell lines to pomalidomide, as evidenced by the decrease in cell viability (**Figure 5A**). We further performed flow cytometry apoptosis assays and observed that glycine promoted the anti-MM effects of pomalidomide in MM cell lines (**Figures 5B, C**), corroborating the results obtained with the CCK-8 assays. These findings suggest that glycine may enhance the sensitivity of MM cells to pomalidomide and could potentially be used as a therapeutic strategy to improve treatment outcomes for pomalidomide-resistant MM patients.

### 3.5 Differences of peripheral immune cell proportion in pomalidomide-resistant group

**Figure 6:**
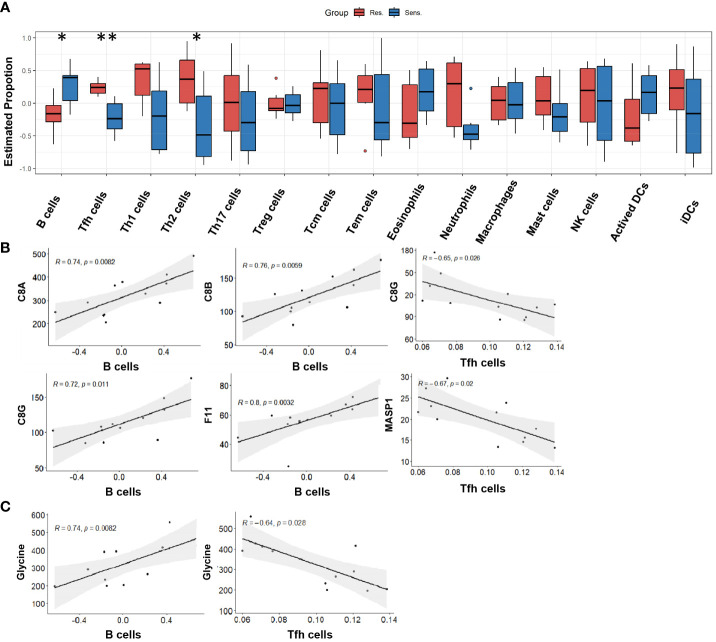
Peripheral immune cell proportion in pomalidomide-resistant group. **(A)** The distribution of cellular proportion of 15 immune cells in the peripheral blood of pomalidomide resistant and sensitive patients. Boxplot shows the median and interquartile range. *P < 0.05, **P < 0.01; Wilcoxon test. **(B)** Correlation among complement components and differential immune cells. *P* < 0.05. **(C)** Correlation among the level of glycine and differential immune cells. *P* < 0.05.

To gain further insight into the immune cell landscape in pomalidomide-resistant and -sensitive MM patients, we utilized the ssGSEA algorithm to estimate the proportions of various immune cell types using the protein expression profiles of reference immune cell types (18, 19). Our analysis revealed a significant decrease in the proportion of B cells and an increase in the proportion of T follicular helper(Tfh) and Th2 cells in the resistant group compared to the sensitive group, suggesting a critical role for these cells in the resistance response to pomalidomide (**Figure 6A**). Correlation analysis between immune cells and differentially expressed complement proteins revealed that B cells negatively correlated with C8A, C8B, C8G, and F11, while Tfh cells positively correlated with C8G and MASP1 (**Figure 6B**). Furthermore, we observed a similar pattern of correlation between immune cell proportions and the level of glycine (**Figure 6C**). These findings suggest that glycine and the complement pathway may work in concert to promote pomalidomide resistance in MM patients.

### 3.6 WGCNA profiling reveals glutathione derivative biosynthetic metabolism associated with lymphocytes in MM patients

**Figure 7:**
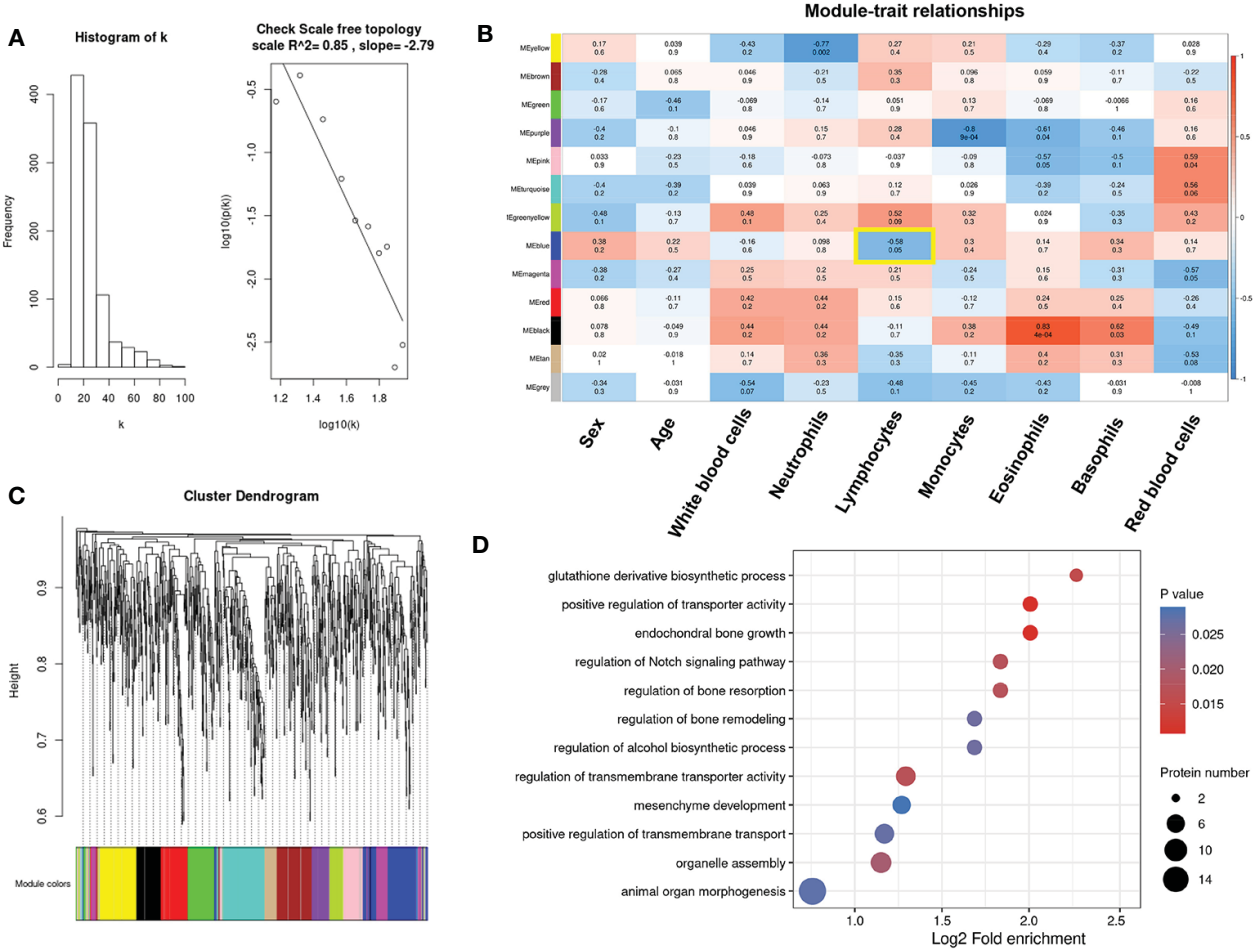
Identification of key modules connected with differential immune cells through weighted gene co-expression network analysis (WGCNA). **(A)**Histogram of connectivity distribution and checking the scale-free topology when β = 5. **(B)** The cluster dendrogram of the proteins. Each branch in the figure represents one protein, and every color below represents one co-expression module. **(C)** Heatmaps of the correlation between module eigengenes and clinical traits. The color of cells in the heatmap represented the correlation coefficients of different sizes. Specifically, red colors represented the positive correlations and blue colors stood for the negative correlations. The corresponding p-value was shown below in parentheses. **(D)** Enrichment analysis of gene ontology for proteins in the blue module.

Immune infiltration analysis revealed that lymphocytes, including B cells, Tfh cells and Th2 cells, were found to play important roles in the resistance response of multiple myeloma patients to pomalidomide. To identify key modules associated with clinical features, we constructed a weighted co-expression network using the protein expression profile. The power of β = 5 (scale free R2 = 0.85) was selected as the soft-thresholding parameter to ensure a scale-free network (**Figures 7A**, **S2**). Using average linkage hierarchical clustering, we identified 13 co-expression modules, with the blue module showing a significant negative correlation with the proportion of lymphocytes (cor = -0.58, p-value = 0.05) (**Figures 7B, C**). Furthermore, GO analyses of the proteins in the blue module revealed significant enrichment in pathways associated with redox balance, such as glutathione derivative biosynthetic process (GO:1901687) and regulation of peroxidase activity (GO:2000468) (**Figure 7D**). Since glutathione is a small molecule composed of glycine, glutamate, and cysteine, the supply of these amino acids dictates glutathione levels. Therefore, consistent with the ssGSEA revealing the correlation between glycine and immune cells, glutathione metabolism significantly correlated with lymphocytes, indicating that glycine could play an important role in the immune reaction related to the resistance response of multiple myeloma patients to pomalidomide.

## 4 Discussion

Drug resistance is a significant obstacle in the treatment of patients with multiple myeloma. The tumor immune microenvironment (TIME), consisting of various immune and stromal cells, is a major contributing factor to tumor relapse and drug resistance (26). Previous proteomic studies on multiple myeloma have focused mainly on molecular changes in myeloma cells, with limited investigation into the TIME (27–30). Here, we employed an integrated proteomics and metabolomics approach to examine plasma samples from pomalidomide-resistant/sensitive patients. Our results demonstrate a significantly lower level of complement components in the resistant group compared to the sensitive group. We found that the expression of complement activating proteins and complement terminal pathway proteins was significantly down-regulated in the drug-resistant group, suggesting that complement deficiency correlates with resistance to pomalidomide.

Patients with multiple myeloma have complex immunological defects (31). As an innate immune surveillance system, complement is a functional bridge between innate and adaptive immune responses, and plays a crucial role in almost every step of the immune reaction (32). Previous studies have shown abnormalities in complement levels and function in multiple myeloma (28, 33), leading to an imbalance in the immune system. Compared to the normal group, the catabolism of C1q in the plasma of myeloma patients was significantly accelerated. Yang et al. revealed that C1q levels are negatively correlated with MM tumor burden (34). A retrospective study showed that low levels of C4 were significantly correlated with poor prognosis in patients with MM (35). Our research suggests that the defect of complement in multiple myeloma should not be explained on the basis of a single complement component abnormality, but rather results from a group of complement abnormalities.

Previous studies have investigated the role of complement in drug resistance in MM. Daratumumab, a monoclonal antibody against CD38, has shown promising anti-tumor activity in the treatment of MM. It has been reported that resistance to daratumumab was associated with the upregulation of complement inhibitory proteins CD55 and CD59 on the MM cells (36). Complement deficiency may be a common feature in the development of resistance to immunomodulatory drugs, as highlighted by our findings.

Our study emphasizes the significance of the complement pathway in the development of drug resistance in MM, although the reasons for the complement deficiency remain unclear. Here we propose potential causes of complement deficiency in myeloma. The matrix metalloproteinases (MMPs) play an important role in tumor progression, including migration, invasion, metastasis and angiogenesis (37–39). Membrane-type 1 matrix metalloproteinase (MT1-MMP) interacts with the C1q and C3b components of the complement system and inactivates the complement propagation cascade, protecting tumor cells from the complement-mediated cytolysis (40, 41). In addition, it has been demonstrated that MT1-MMP and MT2-MMP are highly expressed in MM cells rather than in normal B cells and plasma cells, and contribute to the degradation of the extracellular matrix and the invasion of MM (42). Further experimental work is required to define functional relationships of MMPs with complement contributing to pomalidomide resistance in multiple myeloma.

Furthermore, our targeted metabolomics analysis showed that glycine was significantly down-regulated in pomalidomide-resistant MM patient sera. Our results demonstrate that the addition of glycine increases the sensitivity of MM cell lines to pomalidomide. While glycine has been shown to promote MM proliferation through glutathione synthesis (43, 44), our study found that glycine synergizes with pomalidomide to promote apoptosis and suppress proliferation in MM cells. The consumption and release profiles of the NCI-60 cancer cell lines revealed that glycine has a heterogeneous pattern of consumption and release, which may explain its paradoxical effects on cancer cells (45). In addition, dietary supplementation of glycine has been reported to inhibit the growth of certain types of tumors, such as liver tumors and melanoma tumors (46). Additional experimental investigation is necessary to elucidate the precise mechanisms responsible for the impacts of glycine in MM.

Pomalidomide is a drug known to have a therapeutic effect through various mechanisms, including immune modulation by enhancing the function of T and NK cells (47). However, the contribution of immune cells to pomalidomide resistance remains poorly understood. In this study, we aimed to estimate the proportions of immune cells in peripheral blood and identify potential immune signatures associated with pomalidomide resistance using the ssGSEA algorithm. Our immune infiltration analysis revealed that resistant patients had a lower proportion of B cells and a higher proportion of Tfh cells in peripheral blood compared to the sensitive group, indicating the presence of an underlying immune signature that could predict the likelihood of benefiting from pomalidomide treatment.

B cell lineage is significantly altered in MM, characterized by a disrupted equilibrium towards the excessive proliferation of malignant plasma cells (48). Previous studies have shown that the level of B cells is inversely correlated with disease stage and is reduced in MM patients (49, 50). Our findings are consistent with these previous studies, indicating that the decreased level of B cells in peripheral blood of resistant patients may be indicative of a more heavily compromised B cell lineage. Further research is needed to elucidate the mechanisms behind the involvement of B cells in pomalidomide resistance.

T follicular helper (Tfh) cells play a crucial role in the formation of germinal centers (GCs) by providing necessary signals to B cells for their differentiation into plasmablasts and plasma cells that secrete high-affinity and isotype-switched antibodies (51). Circulating follicular helper T cells (cTfh) in peripheral blood have also been shown to induce plasmablast or plasma cell differentiation and antibody production (52). Resting PD-1+CXCR3− cTfh cells are a population of circulating memory Tfh cells that exhibit similar stimulating activity to GCTfh cells after activation (53), and transcriptionally and clonally resemble GC Tfh cells, suggesting that GCTfh can exit the GC and enter the pool of circulating memory T cells (54, 55). We hypothesize that cTfh share clonotypes with GCTfh cells that are associated with malignant plasma cells, potentially inducing the differentiation of B cells into malignant plasma cells. This proposed mechanism could provide insights into the relapse of multiple myeloma.

Moreover, our correlation analysis revealed significant correlations between B cells and Tfh cells with complement components. It has been known for 40 years that B cells express complement receptor CR2 (CD21), which interacts with C3d and iC3b on the surface of the antigen and induces an increase of B cell receptor (BCR) signaling (56, 57). Additionally, there is considerable evidence indicating that complement regulates T cell proliferation and differentiation (58–60). Our findings support the involvement of the complement pathway in the immune response underlying the resistance to pomalidomide.

Reactive oxygen species (ROS) production is a critical step in immune cell activation, and amino acids play a central role in regulating redox balance within immune cells (24, 61, 62). Glutathione is a major cellular antioxidant molecule in cells (63). Upon activation, T cells increase glutathione synthesis (64), while B cells and macrophages require glutathione to regulate their redox status after ROS production (65, 66). Our WGCNA profiling results are consistent with previous studies, showing that glutathione derivative biosynthetic metabolism is significantly correlated with lymphocyte levels in MM patients. As glycine is a component of glutathione, its availability is a key determinant of glutathione levels. Recent evidence suggests that glycine has immunomodulatory effects (67). We also found that glycine levels were significantly correlated with complement components and immune cell proportions. Our results suggest that glycine may play a potential role in pomalidomide resistance through immunomodulatory effects, which require further investigation. Our analysis also highlighted the potential involvement of T follicular helper (Tfh) cells and B cells in the resistance to pomalidomide. These cells were found to be significantly correlated with complement levels, indicating their potential role in immune response and resistance to pomalidomide.

We acknowledge that this study has some limitations. First, the sample size in this study was relatively small, and we did not have matched samples during treatment or at progression. The results need a larger sample size to verify. Second, the analysis of immune cells proportion is based on the estimation of the ssGSEA algorithm, which needs to be further confirmed by flow cytometry. Finally, this study lacks laboratory validation of the molecular mechanism regulating the resistance of pomalidomide, which can be further explored.

## 5 Conclusions

In summary, our study sheds light on the molecular changes that occur in the plasma of MM patients who are resistant to pomalidomide and highlights the potential involvement of glycine, Tfh cells, and B cells in this resistance mechanism. These findings may provide insights into the development of immune-related therapeutic biomarkers and the identification of resistance mechanisms for immunomodulatory drugs in MM.

## Data availability statement

The original contributions presented in the study are included in the article/**Supplementary Material**. Further inquiries can be directed to the corresponding authors.

## Ethics statement

The studies involving humans were approved by the medical ethics committee of the Shanghai Fourth People’s Hospital. The studies were conducted in accordance with the local legislation and institutional requirements. The participants provided their written informed consent to participate in this study. Ethical approval was not required for the studies on animals in accordance with the local legislation and institutional requirements because only commercially available established cell lines were used.

## Author contributions

YZ: Data curation, Formal Analysis, Investigation, Methodology, Software, Validation, Visualization, Conceptualization, Writing – review & editing. CL: Data curation, Formal Analysis, Investigation, Methodology, Software, Validation, Visualization, Writing – original draft. HJ: Formal Analysis, Methodology, Resources, Software, Writing – review & editing. LL: Resources, Software, Validation, Writing – review & editing. YTZ: Investigation, Resources, Writing – review & editing. WY: Conceptualization, Funding acquisition, Methodology, Project administration, Supervision, Writing – review & editing. WF: Conceptualization, Funding acquisition, Methodology, Project administration, Resources, Software, Validation, Writing – review & editing.
