# Supplementary material for: Multi-omics investigation of the resistance mechanisms of pomalidomide in multiple myeloma
Source: Front Oncol. 2023 Sep 19;13:1264422. doi: 10.3389/fonc.2023.1264422 (PMC10549987; doi:10.3389/fonc.2023.1264422)
Supplement: Supplementary file 1 [file DataSheet_1.docx]

Supplementary Material

Multi-omics investigation of the resistance mechanisms of Pomalidomide in Multiple Myeloma

Yan Zhuang^1,2 †^ , Chenyu Li^1 †^, Hua Jiang^2^ , Lu Li^2^ , Yuanteng Zhang^3^ , Wei Yu^1 *^, WeiJun Fu^2*^

^1^ State Key Laboratory of Genetic Engineering, School of Life Sciences, Fudan University, Shanghai, China

^2^ Department of Hemetology, Shanghai Fourth People’s Hospital, School of Medicine, Tongji Unversity, Shanghai, China

^3^ Institute of Drug Discovery and Design, College of Pharmaceutical Sciences, Zhejiang University, Hangzhou, Zhejiang, China

*** Correspondence:** WeiJun Fu, [fuweijun2010@hotmail.com](mailto:fuweijun2010@hotmail.com); Wei Yu, [yuw@fudan.edu.cn](mailto:yuw@fudan.edu.cn)

Yan Zhuang1,2 † and Chenyu Li1 †, These authors contributed equally to this work and share first authorship

# Supplementary Figures


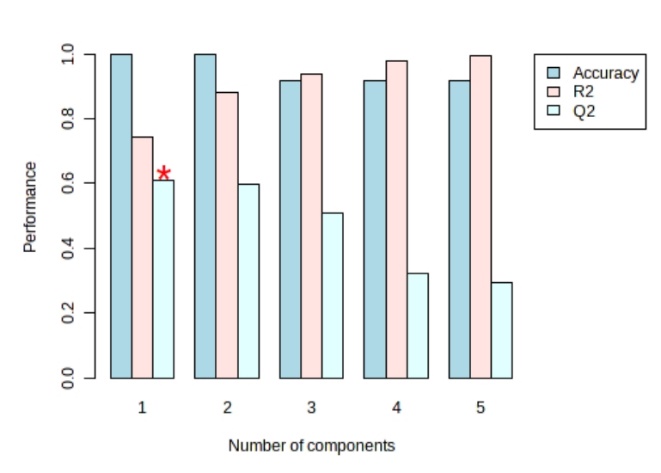


**Supplementary Figure 1. PLS-DA performance measurements.**

Accuracy, multiple correlation coefficient R2 and the explained variance in prediction Q2 are shown. The red asterisk indicates the best value of selected measure (Q2).


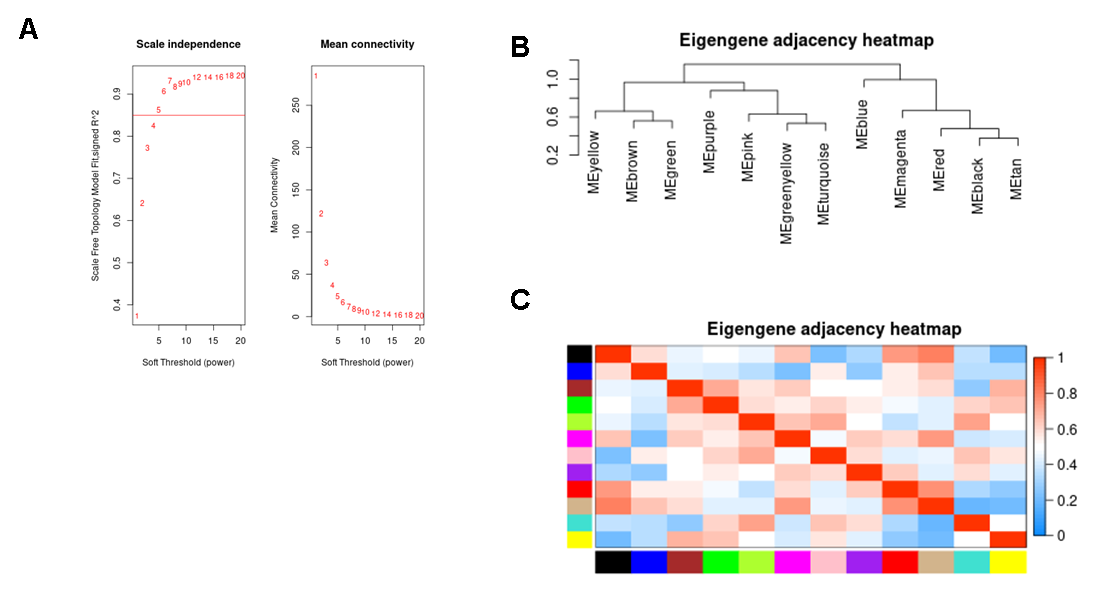


**Supplementary Figure 2. Weighted gene co-expression network analysis**

(A)Analysis of the scale-free fit index and the mean connectivity for various soft-thresholding powers (β). (B)Hierarchical clustering tree for clustering modules. (C) Heatmap of the correlation between identified modules.
